# Definition and Criteria for the Assessment of Expertise in Psychotherapy: Development of the Psychotherapy Expertise Questionnaire (PEQ)

**DOI:** 10.3390/ejihpe13110173

**Published:** 2023-11-02

**Authors:** Alessio Gori, Eleonora Topino, Marco Cacioppo, Adriano Schimmenti, Vincenzo Caretti

**Affiliations:** 1 Department of Health Sciences, University of Florence, Via di San Salvi 12, Pad. 26, 50135 Florence, Italy; 2 Integrated Psychodynamic Psychotherapy Institute (IPPI), Via Ricasoli 32, 50122 Florence, Italy; 3 Department of Human Sciences, LUMSA University of Rome, Via della Traspontina 21, 00193 Rome, Italy; eleonora.topino@gmail.com (E.T.); m.cacioppo@lumsa.it (M.C.); vincenzocaretti@gmail.com (V.C.); 4 Faculty of Human and Social Sciences, UKE—Kore University of Enna, Cittadella Universitaria, 94100 Enna, Italy; adriano.schimmenti@unikore.it

**Keywords:** therapist expertise, professional competence, therapeutic efficacy, therapist skills, training, supervision

## Abstract

Therapist expertise is a complex, multifaceted, and continually evolving concept. Defining this construct and its constituent components can yield a substantial contribution to the field of psychotherapy, consequently enhancing the comprehension of the fundamental factors that underlie its effectiveness. Within this framework, the present research aimed at developing and assessing the psychometric properties of the Psychotherapy Expertise Questionnaire (PEQ), a self-report measure to assess therapist expertise. A sample of 260 psychotherapists of various theoretical orientations were involved in this research. They completed a survey that included the PEQ as well as other self-reported measures aimed at evaluating personality traits, self-efficacy, self-esteem, and insight orientation. The analysis provided evidence of a good fit for both a correlational model with eight factors and a higher-order model, where the eight subdimensions were grouped into subjective (performance; cognitive functioning; personal and relational qualities of the therapist; therapist self-assessment) and objective (experience; reputation with clients and colleagues; training and professional updating; deontological ethics and setting rules) factors. The eight dimensions, two higher-order factors, and total score all showed excellent levels of internal consistency. Furthermore, significant associations were found between PEQ scores and insight orientation, general self-efficacy, self-esteem, personality traits, and time exercising clinical practice. To conclude, the Psychotherapy Expertise Questionnaire (PEQ) is a valuable, theoretically guided, and psychometrically robust self-report measure designed to assess therapist expertise and its constitutive dimensions. This measure can have practical applications in guiding tailored training and customised supervision.

## 1. Introduction

In one of the initial meta-analytic studies examining the effectiveness of psychotherapy, Smith, Glass, and Miller [1] conducted an analysis of 475 research studies comparing the outcomes of individuals who received psychotherapy to those who did not receive any treatment. The results revealed a substantial effect size (ES) of 0.85 (SD = 1.25). This value signifies that, on average, individuals who underwent psychotherapy experienced significantly greater improvements at the conclusion of treatment compared to those who received no intervention in approximately 80% of cases [1]. This pioneering meta-analysis represented the first quantitative estimate of the effectiveness of psychotherapy [2,3], and it was an important starting point in this field, subsequently enriched by abundant further evidence (e.g., [4,5,6]). More recently, Leichsenring and colleagues [7] published an umbrella review and meta-analytic evaluation aiming to evaluate the efficacy of psychotherapy and pharmacotherapy for major mental disorders in adults (depressive disorders, anxiety disorders, post-traumatic stress disorder, obsessive–compulsive disorder, somatoform disorders, eating disorders, attention deficit/hyperactivity disorder, substance use, insomnia, schizophrenia spectrum disorders, and bipolar disorder). The study included 102 meta-analyses and a total of 650,514 patients. The results of this review showed an advantage of psychotherapy and pharmacotherapy in the treatment of mental disorders in adults compared to control conditions. Furthermore, psychotherapy also achieved a greater standardised mean difference in comparison to pharmacotherapy in terms of long-term efficacy for the treatment of post-traumatic stress disorder (PTSD) [7]. This line of research is constantly expanding, and other important evidence details and testifies to the effectiveness of psychotherapy (e.g., [8]).

Within this framework, growing research has focused on therapeutic processes and outcomes, attributing the primary role in therapeutic change to common factors [3,9,10,11]. Related to this, an intriguing inquiry that has surfaced pertains to whether the efficacy of psychotherapy is partially attributable to the abilities of the therapist [12]. Therefore, the topic of the effectiveness of therapists, or better “therapist expertise”, is currently being debated among psychotherapy researchers and experts around the world.

One of the first definitions of expertise in psychotherapy comes from Shanteau [13], who conceptualised the construct as “*increased quality of performance gained with additional experience*” (p. 218) [14]. Other definitions have followed over time. One such definition pertains to the “expert therapist”, who possesses the ability to organise their knowledge hierarchically, focus on what is relevant, and develop functional accounts of the problem; additionally, the expert therapist has the capacity to adjust to new situations and reflect on their knowledge and actions, both generally and in particular situations [15,16]. 

A more integrated and comprehensive definition is given by Hill and colleagues [16], who adopt a performance-based approach, suggesting that therapist expertise exists on a continuum, ranging from highly inexpert to highly expert. They define expertise in the practice of psychotherapy “*…as the manifestation of the highest levels of ability, skill, professional competence, and effectiveness*” (p. 9). [16] These authors describe a multidimensional construct, which can be analysed through a focus on the following dimensions: (1) performance, referring to both technical and relational skills that contribute to interventions and interactions with the client; (2) cognitive processing, referring to the ability to process and organise information useful for the therapeutic process; (3) client outcomes, referring to the results of the therapeutic process observed in terms of client engagement, dropout rates, and therapeutic effects on the client; (4) experience, referring to the extent and quality of activities performed within the specific professional field of psychotherapy; (5) personal and relational qualities of the therapist, referring to aspects that may influence the therapist’s functioning in both personal and relational areas which may affect the therapeutic process and relation; (6) credentials, referring to certifications from accredited institutions in the field of psychotherapy; (7) reputation, referring to expressions of recognition and appreciation for one’s professional work from colleagues and positive feedback received from clients; and (8) therapist self-assessment, referring to the therapist’s self-appraisal about their professional competencies [16]. 

Given the important implications in clinical professional practice of the construct of therapist expertise, several measures have been developed for its evaluation (e.g., the Counselor Evaluation Rating Scale [17] or the ENhancing Assessment of Common Therapeutic factors, [18]) and for the assessment of its peculiar dimensions, such as the ability to structure the therapeutic alliance (e.g., the Working Alliance Inventory [19]). Nonetheless, many of these tools appear limited to some specific domains, and none of them cover all the components identified by Hill and colleagues [16]. Since the topic of therapist expertise is constantly changing and expanding, the field of psychotherapy research may benefit from an updated instrument to assess it. 

Based on the aforementioned considerations, the purpose of this study was to develop the Psychotherapy Expertise Questionnaire (PEQ), a new measure to assess therapist expertise and its subdimensions, referring to and adapting the theoretical model conceptualised by Hill and colleagues [16]. The specific aims were:

(1) The conceptualisation, identification, and selection of PEQ items;
(2) The analysis of the psychometric properties of the PEQ in a sample of Italian psychotherapists;
(3) The investigation of the relationship between the total score and subdimensions of the PEQ with levels of self-esteem, general self-efficacy, insight orientation, personality traits, and time exercising clinical practice.

## 2. Materials and Methods

### 2.1. Participants, Procedure, and Ethics

The research involved a sample of 260 psychotherapists (see Table 1), predominantly females (87.3%), with ages ranging from 29 to 70 years (*M_age_* = 41.17 years; *SD* = 7.80). 

Concerning their professional activity, most of them reported engaging in clinical practice for 5 to 10 years (46.5%). Furthermore, the involved psychotherapists reported various theoretical orientations: the majority referred to the Integrated model (20.4%), followed by the Systemic (17.7%), Psychodynamic (16.9%), Cognitive Behavioural (15.4%), Cognitive (11.5%), Humanistic (9.2%), and, to a lesser extent in this sample, Psychoanalytic (8.8%) models. Participants were volunteers recruited via the Internet through a snowball procedure. They completed the survey hosted on the Google Forms platform after providing electronic informed consent. Before starting, they were also informed about the general aim of this study, and privacy and anonymity were guaranteed. All the procedures of this research were approved by the first author’s institutional Ethical Committee.

### 2.2. Development of the Psychotherapist Expertise Questionnaire (PEQ)

The PEQ was designed as a comprehensive and psychometrically robust measure to assess psychotherapists’ professional expertise. During the construction of the PEQ, special attention was given to:

(a) Creating a versatile measure suitable for psychotherapists with various theoretical orientations, using common language and concepts that professionals from different theoretical models could easily relate to;
(b) Elaborating a tool which may provide functional insights for improving and orienting training and professional development and which allows for monitoring a clinician’s level in different constitutive dimensions of psychotherapist expertise;
(c) Implementing a questionnaire with a strong theoretical foundation, supported by valid empirical evidence concerning this field.

According to these premises, a thorough analysis of the scientific literature on specific components and facets of psychotherapist expertise guided the creation of the PEQ items. Specifically, the criteria for evaluating expertise as conceptualised by Hill and colleagues [16] were adopted as the initial reference. These criteria were integrated, modified, and reconceptualised as subjective (i.e., internal, personal, and/or professional characteristics of the psychotherapist) or objective (i.e., characteristics assessable through external and/or concrete evidence and feedback) factors. The following eight theoretical dimensions were obtained:

- *Subjective factors*
  - *Performance.* Psychotherapeutic practice involves a complex and deliberately established relationship, where change can be fostered [20] and the patient/client can be involved in a corrective relational experience [21]. In line with this, empirical evidence identifies the therapeutic alliance as an important common factor influencing psychotherapy outcomes (see Baier et al. [22] for a systematic review), highlighting the significance of the clinician’s skills not only in establishing and maintaining the alliance [23], but also in the resolution of any potential rupture that may arise (see Eubanks et al. [24] for a meta-analysis). These dynamics also involve the clinician’s ability to collaborate on the goals of therapy (see Tryon et al. [25] for a meta-analysis) and obtain a good level of patient–therapist consent in this regard (see Feinstein et al. [26] for a review), as well as to stimulate high levels of interpersonal coordination [27] with suitable and timely interventions tailored to the client/patient’s needs at that particular moment [28,29].
  - *Cognitive functioning*. The ability to formulate/conceptualise clinical cases, as well as to reflect on the therapeutic process, has been identified in the scientific literature as a relevant factor that can contribute to positive psychotherapy outcomes [30]. These aspects imply the clinician’s ability to gather essential information about the client/patient, effectively manage it, and reason to develop tailored and successful treatment plans [31,32]. This also means that the psychotherapist has acquired the knowledge and competence necessary to identify and manage data relevant to the diagnostic process [33] based on the main international reference systems (e.g., DMS-5-TR [34], ICD-11 [35], PDM-2 [36]).
  - *Personal and relational qualities of the therapist*. An increasing body of evidence highlights how a clinician’s qualities can influence the effectiveness of the psychotherapeutic process [37]. Therefore, it appears relevant for the success of psychotherapy to be a self-aware and non-defensive professional, capable of developing warm, appropriate, functional, and effective connections with their clients/patients [16,38,39]. In this regard, high levels of insight [37], mentalisation [40,41,42], reflexivity [43], and a mindful attitude [44] towards one’s personal vulnerabilities have been shown to be effective in reducing their impact on the therapeutic process and decreasing reactivity in the clinical setting [45,46].
  - *Therapist self-assessment*. Scientific research has highlighted that therapists’ self-rated professional characteristics are associated with their patients’ outcomes (see Heinonen and Nissen-Lie [47] for a review). This suggests that a clinician’s self-perception and attitudes towards therapeutic work can have implications on the quality of the treatment, and feeling confident in one’s professional abilities is a significant factor for therapy to be effective [48,49]. In this regard, previous evidence has shown that therapists’ professional self-confidence and work enjoyment predict stronger early alliances with their clients/patients [50]. Consistently, clinician’s professional self-concept was also associated with realistic definitions of clinical goals and more effective performance [51].
- *Objective factors*
  - *Experience*. Some lines of research have investigated the role of experience in the effectiveness of psychotherapy, highlighting its influence on certain specific outcomes (see Dawson et al. [52] for a review). More specifically, therapists identify formal supervision as one of the most important elements in their professional development [53,54]. Furthermore, greater clinical experience has been associated with a greater sense of mastery in terms of therapeutic work [55], as well as greater integration between personal and professional identities [56]. Finally, previous evidence has demonstrated lower rates of premature discontinuation for more experienced psychotherapists compared to trainees [57].
  - *Reputation with clients and colleagues*. Within academic literature pertaining to this topic, professional reputation has been frequently identified as an indicator of therapist expertise [14,58]. In this regard, clients/patients can be valuable sources of information, since they may or may not recommend the practitioner to other members of the community based on the trust the therapist elicits in them [16]. Indeed, professionals with low levels of expertise exhibit a higher rate of premature termination [57], which is linked to lower symptom improvement [59], greater likelihood of sustained deterioration [60], and, therefore, increased client/patient dissatisfaction [61]. Furthermore, supervisors and colleagues may also provide information about therapists [16], especially if these are based on previous networking experiences and professional collaborations.
  - *Training and professional updating*. Training and continuing professional development are important opportunities and responsibilities for mental health professionals [62]. In this regard, desired results, such as a stronger working alliance, improved client/patient functioning, and more positive perceptions about themselves as therapists, have been found to be significantly associated with training [63,64]. Moreover, previous evidence has shown that time spent in deliberate practice targeted at improving professional skills is a significant predictor of positive therapy outcomes [65]. On the other hand, existing research has also highlighted a drastic drop in knowledge useful to the profession in clinicians who do not engage in lifelong learning [66,67].
  - *Deontological ethics and setting rules*. Mental health professionals are expected to adhere to an ethical code of conduct in the practice of their working activities [68]. Since the establishment of a trusting relationship is a fundamental aspect of psychotherapy [20], the clinician should be guided by principles such as beneficence and non-maleficence, fidelity and responsibility, integrity, justice, and respect for people’s rights and dignity in the exercise of ethical professional practice, to protect both patients and the profession [62,69,70]. In line with this and to further support the argument, scientific evidence highlights that compliance with appropriate setting rules is positively associated with positive therapeutic alliances [71] and the development of therapeutic processes [72], while boundary violations are related to negative outcomes [73,74].

Based on the aforementioned analysis of the scientific literature, the PEQ item pool was generated by the researchers. More specifically, after conceptually identifying the theoretical dimensions that the scale should encompass, a list of associated statements was elaborated (see Table 2). 

This phase was carried out by organising focus groups to enhance the questionnaire’s theoretical consistency and effectiveness, aiming to reduce ambiguity in the items and promote clear and appropriate language related to the topic. Furthermore, a satisfactory agreement on item content was achieved. A 5-point Likert scale response format was adopted, with the following options: 1 = “*not at all*”, 2 = “*a little*”, 3 = “*somewhat*”, 4 = “*much*”, and 5 = “*a great deal*” (see Appendix A). The calculation of total and dimensional scores was conceived as the sum of the scores from corresponding items.

### 2.3. Measures

#### 2.3.1. Psychotherapist Expertise Questionnaire (PEQ)

The *Psychotherapist Expertise Questionnaire* (PEQ) is a 32-item self-report scale used to assess professional expertise in the field of psychotherapy practice. Items (see Appendix A and Table 2) are rated on a 5-point Likert scale, ranging from 1 (*not at all*) to 5 (*a great deal*). The scale was conceptualised to assess four subjective dimensions (performance; cognitive functioning; personal and relational qualities of the therapist; therapist self-assessment), and four objective dimensions (experience; reputation with clients and colleagues; training and professional updating; deontological ethics and setting rules). The calculation of a PEQ total score was conceived through the sum of item scores. In the present sample, the scale showed good indications of internal consistency (for more details on the factor structure and the reliability of the PEQ, refer to the Section 3).

#### 2.3.2. General Self-Efficacy Scale (GSE)

The *General Self-Efficacy Scale* (GSE) [75] is a 10-item self-report scale used to assess general self-efficacy. Items are rated on a 4-point Likert scale, ranging from 1 (*not at all true for me*) to 4 (*very true for me*). A total score ranging from 10 to 40 can be calculated. A higher score indicates greater self-efficacy. The Italian version [76] was used in the present sample and showed excellent indications of internal consistency (*α* = 0.915; *ω* = 0.915).

#### 2.3.3. Rosenberg Self-Esteem Scale (RSES)

The *Rosenberg Self-Esteem Scale* (RSES) [77] is a 10-item self-report scale used to assess general self-esteem. Items are rated on a 4-point Likert scale, ranging from 0 (*strongly agree*) to 3 (*strongly disagree*). A total score ranging from 0 to 30 can be calculated. A higher score indicates greater self-esteem. The Italian version [78] was used in the present sample and showed good indications of internal consistency (*α* = 0.836; *ω* = 0.834).

#### 2.3.4. Insight Orientation Scale (IOS)

The *Insight Orientation Scale* (IOS; originally developed in Italian by Gori et al. [79]) is a 7-item self-report scale used to assess personal orientation toward insight. Items are rated on a 5-point Likert scale, ranging from 1 (*not at all*) to 5 (*a great deal*). A total score ranging from 7 to 35 can be calculated. A higher score indicates greater insight orientation. The original Italian version [79,80] was used in the present sample and showed excellent indications of internal consistency (*α* = 0.906; *ω* = 0.908).

#### 2.3.5. Ten-Item Personality Inventory (TIPI)

The *Ten-Item Personality Inventory* (TIPI) [81] is a 10-item self-report scale designed to assess personality traits. Items are rated on a 7-point Likert scale, ranging from 1 (*disagree strongly*) to 7 (*agree strongly*). Five personality dimensions can be calculated, in line with the Big Five model [82]: extraversion, agreeableness, conscientiousness, neuroticism, and openness. The Italian version [83] was used in the present sample and showed satisfactory indications of internal consistency (extraversion, *α* = 0.747, *ω* = 0.746; agreeableness, *α* = 0.624, *ω* = 0.604; conscientiousness, *α* = 0.659, *ω* = 0.620; neuroticism, *α* = 0.703, *ω* = 0.677; openness *α* = 0.667; *ω* = 0.654).

### 2.4. Data Analysis

**Figure 1:**
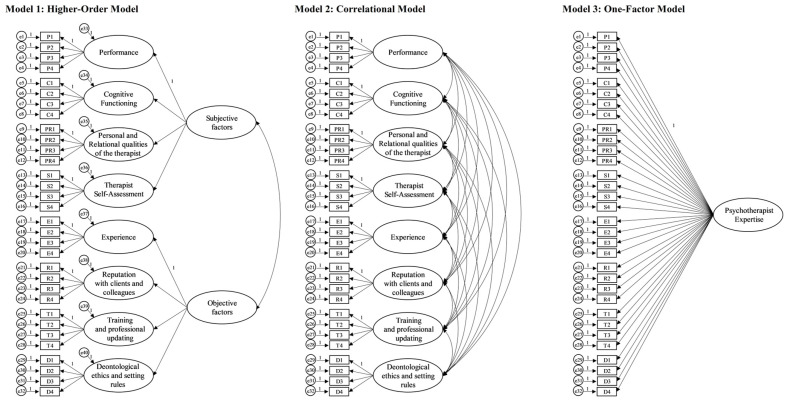
Graphical representation of the tested factor structure models for the PEQ.

The collected data were analysed using SPSS 21.0 (IBM, New York, NY, USA) [84], and AMOS 24.0 (IBM, New York, NY, USA) [85] for Windows. First, descriptive statistics were calculated. Univariate distributions for each item of the PEQ were explored for normality assessment: absolute values of skewness and kurtosis smaller than 1.96 [86] were interpreted as indicative of normal distribution of the sample. Subsequently, the Kaiser–Meyer–Olkin (KMO) statistic and Bartlett’s test of sphericity were applied to assess the suitability of the data for factor analysis: a KMO value above 0.7 and a statistically significant Bartlett’s test (*p* < 0.001) were considered as indicators of appropriateness [87]. Therefore, the hypothesised factor structure of the PEQ was tested utilising confirmatory factor analyses (CFAs). In line with the indications of Kline [88], the following indices were considered to assess the model fit to the data: the chi-square model (*χ^2^*), suggesting a good model fit when the probability value is non-significant (*p* > 0.05) [89]; the comparative fit index (CFI), suggesting a reasonable model fit when the value is above 0.95 [88]; and the root mean square error of approximation (RMSEA) with 90% confidence intervals (from lower confidence interval [LCI] to upper confidence interval [UCI]) and the standardised root mean square residual (SRMR), suggesting a reasonable model fit when the values are less than 0.08 [89,90]. In addition, discrepancy divided by degrees of freedom (CMIN/DF) was also considered, suggesting a reasonable fit for values lower than 5 [91]. Furthermore, three factorial structures were compared through the *Δχ^2^*: (i) a correlational model involving the eight hypothesised factors, (ii) a higher-order model (with the “Subjective” and “Objective” factors as second-order constructs), and (iii) a one-factor solution (see Figure 1). A significant *Δχ^2^* was interpreted as indicative of a statistically significant difference in the fit of the models to the data [92]. Then, information regarding the reliability of the scale was examined through the utilisation of Cronbach’s alpha [93] and McDonald’s omega [94] coefficients, in addition to item–total correlation indices. Finally, Pearson’s r correlation was performed to assess some aspects of concurrent validity.

## 3. Results

Descriptive statistics of the sample are reported in Table 1. Skewness values ranged from |1.151| (item 31) to |0.032| (item 11), and kurtosis values ranged from |0.945| (item 14) to |0.057| (item 19), suggesting a normal distribution. The KMO index of 0.937 and the statistical significance of Bartlett’s test of sphericity (*p* < 0.001) suggested the suitability of the data for factor analysis.

Concerning factor analysis, although all the tested models showed a significant chi-square, the other indices suggested a good fit to the data for both the correlational model (CMIN/DF = 1.957, CFI = 0.903, RMSEA = 0.062 [90% confidence interval: 0.055–0.067], SRMR = 0.057) and the higher-order one (CMIN/DF = 1.999, CFI = 0.903, RMSEA = 0.062 [90% confidence interval: 0.056–0.068], SRMR = 0.057). Finally, the one-factor model, where all 32 items loaded on one factor (see Figure 1), showed a slightly poorer fit: CMIN/DF = 2,651, CFI = 0.836, RMSEA = 0.080 (90% confidence interval: 0.074–0.085), SRMR = 0.061. The exploration of the chi-square variation statistically confirmed the fit superiority of both the higher-order and correlational models compared with the one-factor model (see Table 3). Furthermore, the correlational model also showed a higher statistical fit than the higher-order one.

Focusing on the reliability of the PEQ, item–total correlations (Table 2) ranged from 0.380 (item 29) to 0.758 (item 28), and Cronbach’s alpha and McDonald’s omega coefficients showed good values for both total score (α = 0.90; ω = 0.88) and subscale scores (see Table 4).

Finally, Pearson’s r analysis showed that the PEQ total score and subscale scores were significantly associated with the variables used to assess concurrent validity (see Table 5). Specifically, PEQ total score was significantly and positively correlated with general self-efficacy (*r* = 0.271, *p* < 0.01), self-esteem (*r* = 0.479, *p* < 0.01), insight orientation (*r* = 0.211, *p* < 0.01), and years of clinical practice (*r* = 0.211, *p* < 0.01). Furthermore, significant and positive associations were found between PEQ total score and agreeableness (*r* = 0.276, *p* < 0.01), conscientiousness (*r* = 0.300, *p* < 0.01) and openness (*r* = 0.299, *p* < 0.01), while a significant and negative relationship was found between PEQ total score and neuroticism (*r* = -0.356, *p* < 0.01).

## 4. Discussion

Given the therapist’s role in favouring the positive outcome of the therapeutic process [95,96], the identification and measurement of levels of professional competencies may be particularly relevant for the implementation of optimal training activities and supervisory practice. Therefore, the present research aimed at developing and assessing the psychometric properties of the Psychotherapy Expertise Questionnaire (PEQ), a new measure to assess therapist expertise.

The conceptualisation of the PEQ and the item generation process were theoretically oriented by the recent debates on the definition of the construct and its constituent components (see [16,38,39,97,98,99]). To the best of the authors’ knowledge, the Psychotherapy Expertise Questionnaire (PEQ) is the first self-report measure assessing therapist expertise and its dimensions, by adopting, integrating, and redefining Hill and colleagues’ [16] perspective, all while taking into account contemporary scientific evidence. 

The PEQ showed excellent psychometric properties, with good indications of validity and reliability. The results of the CFA confirmed the statistical goodness of fit of the model involving the eight hypothesised dimensions: *performance; experience; cognitive functioning; reputation with clients and colleagues; personal and relational qualities of the therapist; training and professional updating; therapist self-assessment; and deontological ethics and setting rules* (see Appendix A and Table 2 for the original Italian version and English translation of the items, respectively). Such components were also grouped into two higher-order factors consistent with previous views [100,101]: (1) *Subjective* (performance; cognitive functioning; personal and relational qualities of the therapist; therapist self-assessment), referring to internal, personal, and/or professional characteristics of the psychotherapist; and (2) *Objective* (experience; reputation with clients and colleagues; training and professional updating; deontological ethics and setting rules), referring to characteristics assessable through external and/or concrete evidence and feedback. This categorisation reflects the complexity of the construct of expertise in psychotherapy, involving the ability to effectively implement processes that are not directly and clearly observable or discrete [102]. These processes, on one hand, remain intangible and related to internal perception, but on the other hand, they can be linked to concrete factors, such as the level of training and supervision, as well as external feedback, such as the esteem of patients and colleagues [16,39,100,101]. Although the results (specifically, the *Δχ^2^* test [92]) indicated the statistical superiority of the correlational model, the higher-order model also showed a good fit to the data, supporting the possibility of considering and using the Objective and Subjective factors too. Additionally, the eight dimensions, the two higher-order factors, and the total score all demonstrate excellent levels of internal consistency. These findings may suggest that, despite the complexity, breadth, and multidimensionality of the construct of therapist expertise [103], the items of the PEQ effectively measure different facets of the same phenomenon.

This was further supported by the association between the PEQ and the measures used to assess concurrent validity, namely insight orientation, self-efficacy, and, to a greater extent, self-esteem. Such data align with prior evidence that emphasises how a stronger inclination towards self-reflection and a positive self-concept may correlate with increased effectiveness in the professional domain [43,104], highlighting the significance of a therapist’s cross-situational characteristics (i.e., those describing the clinician beyond their professional roles) for a successful psychotherapeutic practice (see Heinonen and Nissen-Lie [47] for a review). Furthermore, the PEQ total score, the higher-order factors, and most of the subdimensions also showed significant and positive associations with the extraversion, agreeableness, conscientiousness, and openness personality traits. On the other hand, all the PEQ scores significantly and negatively correlated with neuroticism, in line with previous evidence highlighting that clinicians with higher levels of this trait tend to report more problems in therapy [105]. Consistently, longitudinal research [106] found that neuroticism predicted the therapist’s stressful involvement (e.g., negative reactions to clients, frequent difficulties in practice, and avoidant coping) during psychotherapy. Finally, the PEQ total score, the higher-order factors, and most of the subdimensions were significantly and positively associated with time exercising the clinical practice. Interestingly, the subdimension “*reputation with clients and colleagues*” was an exception and showed no significant association. This result offers valuable insights that contribute to an open debate in the scientific literature within the field. Indeed, while some authors have found no significant effects of years of practice on patient outcomes [107,108,109,110], in other studies, positive therapy results presented a positive association with the clinician’s experience (see Dawson et al. [52] for a review). This information, which deserves further investigation in future research, may enrich the controversial topic concerning therapist experience [52,107,108,109,110,111], suggesting that it is not merely time per se, but rather the quality and quantity of clinical practice that drive more positive outcomes, greater client satisfaction, and an enhanced reputation. This aligns with the significant and positive association between two sub-dimensions of the PEQ, namely “*experience*” and “*reputation with clients and colleagues*”.

### 4.1. Practical Implications 

Therapist expertise is a multifaceted, expansive, and perpetually evolving concept of great significance in comprehending therapeutic effectiveness [103]. The definition of measurement criteria and the development of assessment instruments hold substantial practical implications and favour knowledge advances in this field. Indeed, the results that may be provided by using the PEQ can be harmoniously integrated with previous research that explores various essential elements affecting psychotherapist effectiveness (e.g., the emotional variables of the therapist or the facilitators of patient engagement with therapy, to name a few [112,113]), favouring further advances toward a more comprehensive understanding of the therapeutic process. Moreover, being aware of the dimensions that underlie therapist expertise could help therapists monitor themselves and improve outcomes through thoughtful and deliberate practice, thus enhancing the sense of professional self-efficacy [114,115,116]. In the educational domain, delineating the desired attributes for therapists could inspire training, and their measurement could be useful for orienting and defining programs tailored to each specific professional [117]. Furthermore, the definition and assessment of the components of therapeutic expertise could support supervisory practice, which could be aimed at implementing the cultivation of the less developed dimensions [118]. In conclusion, more accurate self-monitoring, training, and supervision can enhance the effectiveness of psychotherapeutic practice, leading to a ripple effect of positive outcomes for public health.

### 4.2. Limitations and Suggestions for Future Research

In addition to the insights gained from this study, its limitations should be recognised and avenues for future research in this domain should be explored. First, snowball sampling was implemented to gather participant data, which may have introduced potential biases due to its non-random nature. A randomised sampling method could be employed in future research to mitigate this limit. Furthermore, participants were predominantly women, and this may affect the generalisability of the results to more balanced samples. Although this aligns with the gender distribution observed among mental health professionals in many countries (e.g., [119,120,121]), deliberate efforts to recruit a more balanced sample could enhance the applicability of the results in future research. Moreover, only self-report measures were used, which may have introduced response bias and limit the depth of data collected. For example, social desirability bias could plausibly affect responses to questions related to deontological ethics and rule-setting. The analyses conducted in this study confirm the normal distribution of all the items presented, thus suggesting the absence of this issue. Nevertheless, in future research, integrating supplementary objective assessment methods, such as behavioural observations or clinical evaluations, could offer a more comprehensive and equitable insight into the phenomena being investigated. In line with this point, also including the therapist’s perception of patient engagement [122,123,124] or patients’ assessment of the clinician’s expertise for analysing its alignment with that of the therapist could be useful suggestions for future research. This multimethod approach could also imply the use of semi-structured interviews aimed at investigating the perceived relationship between the specific constituent elements of therapist expertise and the effectiveness of the therapeutic process, opening new avenues for further future research. Finally, no investigation was conducted to analyse whether therapists’ self-descriptions concerned their clinical work in general or related to therapeutic practice with a specific patient. Exploring this aspect in future research could offer valuable insights, potentially expanding the range of applications for the PEQ.

## 5. Conclusions

The Psychotherapist Expertise Questionnaire (PEQ) is a theoretically grounded and psychometrically robust self-report measure designed to assess psychotherapists expertise and its constitutive dimensions. It was conceptualised by adopting, integrating, and redefining Hill and colleagues’ [16] perspective based on new contemporary scientific evidence, and consists of items elaborated in a language suitable for psychotherapists with various theoretical orientations. Since a growing body of research has found that therapist effectiveness explains a significant portion of the variability in treatment outcomes (see Johns et al. [125] for a review), the PEQ may be a valuable instrument to examine the factors that distinguish some therapists as more effective than others, with practical implications in orienting the training and supervision of mental health professionals.

## Figures and Tables

**Table 1:** Demographic and professional characteristics of the sample (n = 260 psychotherapists).

Characteristics		M ± SD	n	%
Age		41.17 ± 7.80		
Sex				
	Males		33	12.7
	Females		227	87.3
Years of Clinical Practice				
	Less than a year		10	3.8
	1–2 years		50	19.2
	2–5 years		79	30.4
	5–10 years		121	46.5
	More than 10 years		10	3.8
Theoretical Orientation				
	Psychoanalytic		23	8.8
	Psychodynamic		44	16.9
	Cognitive		30	11.5
	Cognitive Behavioural		40	15.4
	Humanistic		24	9.2
	Integrated		53	20.4
	Systemic			

**Table 2:** PEQ items and corresponding dimensions and higher-order factors.

Dimension	Item ^a^
Performance ^b^	1. I am able to wait for the opportune moment to propose a clinical intervention
	2. I am able to repair ruptures that arise in the therapeutic relationship with patients/clients
	3. I am able to effectively share the objectives of the therapeutic work
	4. I am able to establish the therapeutic alliance
Experience ^c^	5. During my training I completed a number of hours of supervision that I consider adequate
	6. I have a consolidated clinical experience
	7. In my clinical practice, I believe I have treated a considerable number of patients/clients
	8. I have treated a diversified clinical caseload
Cognitive Functioning ^b^	9. I can catch the nuances within the therapeutic process
	10. I can make appropriate diagnostic evaluation
	11. I can adequately reflect on clinical cases
	12. I can process and organize the various information that constitutes a therapeutic intervention
Reputation with Clients and Colleagues ^c^	13. I generally get positive feedback from my patients/clients
	14. I have a good reputation for my work as a psychotherapist
	15. Generally, I have adequately rapport with colleagues
	16. In my clinical experience, few patients have prematurely terminated treatment
Personal and Relational Qualities of the Therapist ^b^	17. I am capable of regulating my emotional reactions towards others
	18. I am able to manage my vulnerabilities within the clinical setting
	19. I am attentive about avoiding projections onto patients/clients
	20. I analysed and understood my personal fragility components
Training and Professional Updating ^c^	21. I can effectively integrate advice and feedback from colleagues into my clinical practice
	22. I keep myself updated on the scientific literature related to the profession of psychotherapist
	23. I participated in numerous conferences on topics concerning the profession of psychotherapist
	24. I have completed a period of training that I consider adequate
Therapist Self-Assessment ^b^	25. I consider my clinical work to be satisfactory
	26. I am suitable for the job of psychotherapist
	27. I feel capable of performing the job of psychotherapist
	28. I am satisfied with my therapeutic competence
Deontological Ethics and Setting Rules ^c^	29. I respect the rules of the clinical setting
	30. I am attentive to matters pertaining to ethics and professional deontology
	31. My patients/clients can trust me regarding the protection of professional confidentiality
	32. I am careful to protect the rights of my patients/clients

**Note:** ^a^ English translation of the items from the original version (in Italian). ^b^ Subjective dimension. ^c^ Objective dimension.

**Table 3:** Fit statistics of the PEQ and chi-square variation test.

*Model*	*χ^2^*	*df*	*p*	*CFI*	*RMSEA* (CI)	*SRMR*	*Model Comparison*	*Δχ^2^*	*Δdf*	*p*
Higher-Order Model	877.767	439	<0.001	0.903	0.062 (0.056–0.068)	0.057				
							-	-	-	-
Correlational Model	825.834	422	<0.001	0.911	0.061 (0.055–0.067)	0.054				
							M1–M2	51.933	17	<0.001
One-Factor Model	1192.958	450	<0.001	0.836	0.080 (0.074–0.085)	0.061				
							M1–M3	367.124	28	<0.001
							M2–M3	315.191	11	<0.001

***Note:*** *χ^2^* = chi-square value of model fit; *df* = degree of freedom; CFI = comparative fit index; RMSEA = root mean square error of approximation; SRMR = standardised root mean square residual; *Δχ^2^* = difference in *χ^2^* values between the compared models; *Δdf* = difference in number of degrees of freedom between the compared models; M1 = higher-order model; M2 = correlational model; M3 = one-factor model; CI = RMSEA 90% confidence intervals (from lower confidence interval [LCI] to upper confidence interval [UCI]).

**Table 4:** Indications of internal consistency for PEQ scores and subscores and intra- and inter-factor correlations.

*Score*			*α*	*ω*	*Intra- and Inter-Factor Correlations*									
*Total*	*Higher-Order Factor*	*Dimension*			*1*	*2*	*2.1*	*2.2*	*2.3*	*2.4*	*3*	*3.1*	*3.2*	*3.3*
1. PEQ			0.950	0.950	1									
	2. Subjective		0.930	0.930	0.961	1								
		2.1 *Performance*	0.758	0.762	0.875	0.904	1							
		2.2 *Cognitive functioning*	0.831	0.837	0.868	0.887	0.744	1						
		2.3 *Personal and Relational qualities of the therapist*	0.748	0.746	0.747	0.814	0.693	0.631	1					
		2.4 *Therapist Self-Assessment*	0.897	0.902	0.849	0.874	0.730	0.700	0.560	1				
	3. Objective		0.889	0.889	0.962	0.850	0.780	0.784	0.624	0.759	1			
		3.1 *Experience*	0.813	0.829	0.840	0.746	0.668	0.710	0.508	0.692	0.869	1		
		3.2 *Reputation with clients and colleagues*	0.742	0.745	0.828	0.758	0.700	0.674	0.547	0.705	0.833	0.645	1	
		3.3 *Training and professional updating*	0.724	0.746	0.747	0.618	0.560	0.594	0.453	0.538	0.817	0.612	0.537	1
		3.4 *Deontological ethics and setting rules*	0.741	0.736	0.682	0.615	0.597	0.531	0.535	0.492	0.696	0.410	0.531	0.474

***Note:*** All the correlations were significant at the 0.01 level (2-tailed).

**Table 5:** Correlation between PEQ scales administered for concurrent validity.

	PEQ Total Score	Subjective Score	Performance	Cognitive Functioning	Personal and Relational Qualities of the Therapist	Therapist Self-Assessment	Objective Score	Experience	Reputation with Clients and Colleagues	Training and Professional Updating	Deontological Ethics and Setting Rules
General Self-Efficacy	0.271 **	0.298 **	0.235 **	0.268 **	0.201 **	0.318 **	0.223 **	0.198 **	0.249 **	0.145 *	0.119
Self-Esteem	0.479 **	0.540 **	0.446 **	0.449 **	0.376 **	0.581 **	0.383 **	0.433 **	0.365 **	0.183 **	0.209 **
Insight Orientation	0.221 **	0.251 **	0.203 **	0.231 **	0.212 **	0.226 **	0.175 **	0.134 *	0.177 **	0.109	0.157*
Extraversion	0.186 **	0.224 **	0.169 **	0.255 **	0.207 **	0.150 *	0.136 *	0.118	0.193 **	0.054	0.071
Agreeableness	0.276 **	0.287 **	0.310 **	0.216 **	0.217 **	0.259 **	0.245 **	0.239 **	0.243 **	0.121	0.181 **
Conscientiousness	0.300 **	0.306 **	0.259 **	0.308 **	0.252 **	0.245 **	0.272 **	0.199 **	0.222 **	0.230 **	0.249 **
Neuroticism	−0.356 **	−0.382 **	−0.363 **	−0.330 **	−0.319 **	−0.321 **	−0.304 **	−0.278 **	−0.294 **	−0.192 **	−0.211 **
Openness	0.299 **	0.288 **	0.240 **	0.311 **	0.213 **	0.234 **	0.287 **	0.253 **	0.250 **	0.234 **	0.183 **
Time Exercising	0.245 **	0.205 **	0.236 **	0.124 *	0.166 **	0.191 **	0.266 **	0.447 **	0.121	0.129 *	0.055

***Note***: Bold values indicate significant *p* values. **. Correlation is significant at the 0.01 level (2-tailed). *. Correlation is significant at the 0.05 level (2-tailed).

## Data Availability

The data presented in this study are available on request from the corresponding author. The data are not publicly available due to privacy reasons.

## Appendix A. Psychotherapist Expertise Questionnaire (PEQ)

A seguire sono elencate brevi affermazioni che riguardano il modo di essere nella relazione terapeutica. Il questionario prevede un tipo di formato di risposta con diversi gradi di scelta in modo da cogliere al meglio le sfumature. In questo caso potrai rispondere scegliendo una delle seguenti risposte:

Per niente	Un po’	Abbastanza	Molto	Moltissimo
**1**	**2**	**3**	**4**	**5**

Non esistono risposte giuste o sbagliate; è importante rispondere in maniera onesta. Ti consigliamo di rispondere alle domande una dopo l’altra seguendo l’ordine.

1. Sono in grado di attendere il momento giusto per proporre un intervento clinico	1	2	3	4	5
2. Sono in grado di riparare le rotture che si presentano nella relazione terapeutica con i pazienti/clienti	1	2	3	4	5
3. Sono in grado di condividere adeguatamente gli obiettivi del lavoro terapeutico	1	2	3	4	5
4. Sono in grado di costruire l’alleanza terapeutica	1	2	3	4	5
5. Nella mia formazione ho svolto un numero di ore di supervisione che reputo adeguato	1	2	3	4	5
6. Ho un’esperienza clinica consolidata	1	2	3	4	5
7. Nel mio lavoro clinico credo di aver trattato un numero considerevole di pazienti/clienti	1	2	3	4	5
8. Ho trattato una casistica clinica diversificata	1	2	3	4	5
9. Riesco a cogliere le sfumature nel processo terapeutico	1	2	3	4	5
10. Riesco a formulare delle valutazioni diagnostiche adeguate	1	2	3	4	5
11. So riflettere adeguatamente sui casi clinici	1	2	3	4	5
12. Riesco a elaborare e organizzare le diverse informazioni che compongono un intervento terapeutico	1	2	3	4	5
13. Generalmente ricevo dei feedback positivi dai miei pazienti/clienti	1	2	3	4	5
14. Ho una buona reputazione per quanto riguarda il mio lavoro di psicoterapeuta	1	2	3	4	5
15. Generalmente, mi relaziono in maniera adeguata con i colleghi	1	2	3	4	5
16. Nella mia esperienza clinica pochi pazienti hanno abbandonato prematuramente il trattamento	1	2	3	4	5
17. Sono in grado di regolare le mie reazioni emotive nei confronti dell’altro	1	2	3	4	5
18. So controllare le mie fragilità all’interno del setting clinico	1	2	3	4	5
19. Sono attenta/o a non effettuare proiezioni sui pazienti/clienti	1	2	3	4	5
20. Ho analizzato e compreso le mie componenti di fragilità personale	1	2	3	4	5
21. Sono in grado di integrare in modo efficace nella mia partica clinica i consigli e i feedback dei colleghi	1	2	3	4	5
22. Mi aggiorno sulla letteratura scientifica che riguarda la professione di psicoterapeuta	1	2	3	4	5
23. Ho partecipato a numerosi convegni sui temi che riguardano la professione di psicoterapeuta	1	2	3	4	5
24. Ho svolto un periodo di training formativo che ritengo adeguato	1	2	3	4	5
25. Valuto come soddisfacente il mio lavoro clinico	1	2	3	4	5
26. Sono adatta/o a svolgere il lavoro di psicoterapeuta	1	2	3	4	5
27. Mi sento in grado di svolgere il lavoro di psicoterapeuta	1	2	3	4	5
28. Sono soddisfatta/o della mia competenza terapeutica	1	2	3	4	5
29. Rispetto le regole del setting clinico	1	2	3	4	5
30. Sono attenta/o ai temi che riguardano l’etica e la deontologia professionale	1	2	3	4	5
31. I miei pazienti/clienti possono fidarsi di me rispetto alla tutela del segreto professionale	1	2	3	4	5
32. Sono attenta/o a tutelare i diritti dei miei pazienti/clienti	1	2	3	4	5

## References

[1] Smith M.L., Glass G.V., Miller T.I. (1980). The Benefits of Psychotherapy.

[2] Lambert M.J., Ogles B.M., Norcross J.C., VandenBos G.R., Freedheim D.K., Olatunji B.O. (2016). Treatment outcome studies. APA Handbook of Clinical Psychology: Theory and Research.

[3] Lambert M.J. (2013). The Efficacy and Effectiveness of Psychotherapy. Bergin and Garfield’s Handbook of Psychotherapy and Behavior Change.

[4] Lambert M.J. (2005). Early response in psychotherapy: Further evidence for the importance of common factors rather than “placebo effects”. J. Clin. Psychol..

[5] Leichsenring F., Rabung S. (2008). Effectiveness of long-term psychodynamic psychotherapy: A meta-analysis. JAMA.

[6] Cristea I.A., Gentili C., Cotet C.D., Palomba D., Barbui C., Cuijpers P. (2017). Efficacy of psychotherapies for borderline personality disorder: A systematic review and meta-analysis. JAMA Psychiatry.

[7] Leichsenring F., Steinert C., Rabung S., Ioannidis J.P. (2022). The efficacy of psychotherapies and pharmacotherapies for mental disorders in adults: An umbrella review and meta-analytic evaluation of recent meta-analyses. World Psychiatry.

[8] Leichsenring F., Abbass A., Heim N., Keefe J.R., Kisely S., Luyten P., Steinert C. (2023). The status of psychodynamic psychotherapy as an empirically supported treatment for common mental disorders–an umbrella review based on updated criteria. World Psychiatry.

[9] Karson M., Fox J. (2010). Common skills that underlie the common factors of successful psychotherapy. Am. J. Psychother..

[10] Wampold B.E., Rousmaniere T.G., Goodyear R.K., Miller S.D., Wampold B.E. (2017). What should we practice? A contextual model for how psychotherapy works. The Cycle of Excellence: Using Deliberate Practice to Improve Supervision and Training.

[11] Hill C.E., Norcross J.C. (2023). Psychotherapy Skills and Methods That Work.

[12] Barkham M., Lutz W., Lambert M.J., Saxon D., Castonguay L.G., Hill C.E. (2017). Therapist Effects, Effective Therapists, and the Law of Variability. How and Why Are Some Therapists Better than Others? Understanding Therapist Effects.

[13] Shanteau J. (1992). Competence in Experts: The Role of Task Characteristics. Organ. Behav. Hum. Decis. Processes.

[14] Tracey T.J.G., Wampold B.E., Lichtenberg J.W., Goodyear R.K. (2014). Expertise in Psychotherapy: An Elusive Goal?. Am. Psychol..

[15] Oddli H.W., Halvorsen M.S., Ronnestad M.M. (2014). Expertise Demonstrated. What Does It Mean to Be an Expert Psychotherapist?. https://societyforpsychotherapy.org/expertise-demonstrated/.

[16] Hill C.E., Spiegel S.B., Hoffman M.A., Kivlighan D.M., Gelso C.J. (2017). Therapist expertise in psychotherapy revisited. Couns. Psychol..

[17] Jones L.K. (1974). The Counselor Evaluation Rating Scale: A valid criterion of counselor effectiveness?. Couns. Educ. Supervis..

[18] Kohrt B.A., Jordans M.J., Rai S., Shrestha P., Luitel N.P., Ramaiya M.K., Patel V. (2015). Therapist competence in global mental health: Development of the ENhancing Assessment of Common Therapeutic factors (ENACT) rating scale. Behav. Res. Ther..

[19] Horvath A.O., Greenberg L.S. (1989). Development and validation of the Working Alliance Inventory. J. Couns. Psychol..

[20] Ford D.H., Urban H.B. (1998). Contemporary Models of Psychotherapy: A Comparative Analysis.

[21] Tschacher W., Junghan U.M., Pfammatter M. (2014). Towards a Taxonomy of Common Factors in Psychotherapy—Results of an Expert Survey. Clin. Psychol. Psychother..

[22] Baier A.L., Kline A.C., Feeny N.C. (2020). Therapeutic Alliance as a Mediator of Change: A Systematic Review and Evaluation of Research. Clin. Psychol. Rev..

[23] Shelef K., Diamond G.M., Diamond G.S., Liddle H.A. (2005). Adolescent and Parent Alliance and Treatment Outcome in Multidimensional Family Therapy. J. Consult. Clin. Psychol..

[24] Eubanks C.F., Muran J.C., Safran J.D. (2018). Alliance rupture repair: A meta-analysis. Psychotherapy.

[25] Tryon G.S., Birch S.E., Verkuilen J. (2018). Meta-Analyses of the Relation of Goal Consensus and Collaboration to Psychotherapy Outcome. Psychotherapy.

[26] Feinstein R., Heiman N., Yager J. (2015). Common factors affecting psychotherapy outcomes: Some implications for teaching psychotherapy. J. Psychiatr. Pract..

[27] Wiltshire T.J., Philipsen J.S., Trasmundi S.B., Jensen T.W., Steffensen S.V. (2020). Interpersonal Coordination Dynamics in Psychotherapy: A Systematic Review. Cogn. Ther. Res..

[28] Tschacher W., Haken H., Kyselo M. (2015). Alliance: A Common Factor of Psychotherapy Modeled by Structural Theory. Front. Psychol..

[29] Bonanno G.A., Burton C.L. (2013). Regulatory Flexibility: An Individual Differences Perspective on Coping and Emotion Regulation. Perspect. Psychol. Sci..

[30] Eells T.D. (2022). Handbook of Psychotherapy Case Formulation.

[31] Rainforth M., Laurenson M. (2014). A Literature Review of Case Formulation to Inform Mental Health Practice. J. Psychiatr. Ment. Health Nurs..

[32] Frank R.I., Davidson J. (2014). The Transdiagnostic Road Map to Case Formulation and Treatment Planning: Practical Guidance for Clinical Decision Making.

[33] Bornstein R.F. (2017). Evidence-Based Psychological Assessment. J. Pers. Assess..

[34] American Psychiatric Association (2022). Diagnostic and Statistical Manual of Mental Disorders.

[35] World Health Organization (2019). International Statistical Classification of Diseases and Related Health Problems.

[36] Lingiardi V., McWilliams N. (2017). Psychodynamic Diagnostic Manual: PDM-2.

[37] Castonguay L.G., Hill C.E. (2017). How and Why Are Some Therapists Better than Others?: Understanding Therapist Effects.

[38] Norcross J.C., Karpiak C.P. (2017). Our Best Selves: Defining and Actualizing Expertise in Psychotherapy. Couns. Psychol..

[39] Hill C.E., Hoffman M.A., Kivlighan D.M., Spiegel S.B., Gelso C.J. (2017). Therapist expertise: The debate continues. Couns. Psychol..

[40] Allen J.G., Fonagy P., Bateman A.W. (2008). Mentalizing in Clinical Practice.

[41] Gori A., Arcioni A., Topino E., Craparo G., Lauro Grotto R. (2021). Development of a new measure for assessing mentalizing: The multidimensional mentalizing questionnaire (MMQ). J. Pers. Med..

[42] Gori A., Topino E. (2023). Exploring and deepening the facets of mentalizing: The integration of network and factorial analysis approaches to verify the psychometric properties of the Multidimensional Mentalizing Questionnaire (MMQ). Int. J. Environ. Res. Public Health.

[43] American Psychological Association (2012). Revised Competency Benchmarks in Professional Psychology.

[44] Ryan A., Safran J.D., Doran J.M., Muran J.C. (2012). Therapist Mindfulness, Alliance and Treatment Outcome. Psychother. Res..

[45] Bruce N.G., Manber R., Shapiro S.L., Constantino M.J. (2010). Psychotherapist Mindfulness and the Psychotherapy Process. Psychother. Theory Res. Pract. Train..

[46] Keane A. (2014). The influence of therapist mindfulness practice on psychotherapeutic work: A mixed-methods study. Mindfulness.

[47] Heinonen E., Nissen-Lie H.A. (2020). The professional and personal characteristics of effective psychotherapists: A systematic review. Psychother. Res..

[48] Hatcher R.L., Fouad N.A., Grus C.L., Campbell L.C., McCutcheon S.R., Leahy K.L. (2013). Competence benchmarks: Practical steps toward a culture of competence. Train. Educ. Prof. Psychol..

[49] Knapp S., Gottlieb M.C., Handelsman M.M. (2017). Self-awareness questions for effective psychotherapists: Helping good psychotherapists become even better. Pract. Innov..

[50] Heinonen E., Lindfors O., Härkänen T., Virtala E., Jääskeläinen T., Knekt P. (2014). Therapists’ professional and personal characteristics as predictors of working alliance in short-term and long-term psychotherapies. Clin. Psychol. Psychother..

[51] Reese R.J., Aldarondo F., Anderson C.R., Lee S.J., Miller T.W., Burton D. (2009). Telehealth in Clinical Supervision: A Comparison of Supervision Formats. J. Telemed. Telecare.

[52] Dawson G.C. (2018). Years of clinical experience and therapist professional development: A literature review. J. Contemp. Psychother..

[53] Orlinsky D.E., Botermans J.F., Rønnestad M.H. (2001). Towards an Empirically Grounded Model of Psychotherapy Training: Four Thousand Therapists Rate Influences on Their Development. Aust. Psychol..

[54] Wheeler S., Richards K. (2007). The Impact of Clinical Supervision on Counsellors and Therapists, Their Practice and Their Clients. A Systematic Review of the Literature. Couns. Psychother. Res..

[55] Orlinsky D., Rønnestad M.H., Ambühl H., Willutzki U., Botersman J.-F., Cierpka M., Davis J., Davis M. (1999). Psychotherapists’ Assessments of Their Development at Different Career Levels. Psychother. Theory Res. Pract. Train..

[56] Rønnestad M.H., Skovholt T.M. (2003). The Journey of the Counselor and Therapist: Research Findings and Perspectives on Professional Development. J. Career Dev..

[57] Swift J.K., Greenberg R.P. (2012). Premature Discontinuation in Adult Psychotherapy: A Meta-Analysis. J. Consult. Clin. Psychol..

[58] Norcross J.C., Bike D.H., Evans K.L. (2009). The Therapist’s Therapist: A Replication and Extension 20 Years Later. Psychother. Theory Res. Pract. Train..

[59] Cahill J., Barkham M., Hardy G., Rees A., Shapiro D.A., Stiles W.B., Macaskill N. (2003). Outcomes of patients completing and not completing cognitive therapy for depression. Br. J. Clin. Psychol..

[60] Barrett M.S., Chua W.-J., Crits-Christoph P., Gibbons M.B.C., Thompson D. (2008). Early Withdrawal from Mental Health Treatment: Implications for Psychotherapy Practice. Psychother. Theory Res. Pract. Train..

[61] Björk T., Björk C., Clinton D., Sohlberg S., Norring C. (2009). What Happened to the Ones Who Dropped Out? Outcome in Eating Disorder Patients Who Complete or Prematurely Terminate Treatment. Eur. Eat. Disord. Rev..

[62] American Psychological Association (2016). Revision of Ethical Standard 3.04 of the “Ethical Principles of Psychologists and Code of Conduct” (2002, as Amended 2010). Am. Psychol..

[63] Hilsenroth M.J., Kivlighan D.M., Slavin-Mulford J. (2015). Structured Supervision of Graduate Clinicians in Psychodynamic Psychotherapy: Alliance and Technique. J. Couns. Psychol..

[64] Hill C.E., Baumann E., Shafran N., Gupta S., Morrison A., Rojas A.E., Gelso C.J. (2015). Is Training Effective? A Study of Counseling Psychology Doctoral Trainees in a Psychodynamic/Interpersonal Training Clinic. J. Couns. Psychol..

[65] Chow D.L., Miller S.D., Seidel J.A., Kane R.T., Thornton J.A., Andrews W.P. (2015). The role of deliberate practice in the development of highly effective psychotherapists. Psychotherapy.

[66] Neimeyer G.J., Taylor J.M., Cox D.R. (2012). On hope and possibility: Does continuing professional development contribute to ongoing professional competence?. Prof. Psychol. Res. Pract..

[67] Neimeyer G.J., Taylor J.M., Orwig J.P. (2013). Do continuing education mandates matter? An exploratory study of the relationship between CE regulations and disciplinary actions. Prof. Psychol. Res. Pract..

[68] Ford G.G. (2006). Ethical Reasoning for Mental Health Professionals.

[69] American Psychological Association (2002). Ethical Principles of Psychologists and Code of Conduct. Am. Psychol..

[70] American Psychological Association (2010). Ethical Principles of Psychologists and Code of Conduct (2002, Amended June 1, 2010). http://www.apa.org/ethics/code/index.aspx.

[71] Dambi J.M., Mavhu W., Beji-Chauke R., Kaiyo-Utete M., Mills R., Shumba R., Chibanda D. (2023). The impact of working alliance in managing youth anxiety and depression: A scoping review. npj Mental Health Res..

[72] Anagnostaki L., Zaharia A., Matsouka M. (2017). Discussing the Therapeutic Setting in Child and Adolescent Psychoanalytic Psychotherapy. J. Child Psychother..

[73] Simon R.I. (1992). Treatment Boundary Violations: Clinical, Ethical, and Legal Considerations. J. Am. Acad. Psychiatry Law Online.

[74] Zur O. (2007). Boundaries in Psychotherapy: Ethical and Clinical Explorations.

[75] Schwarzer R., Jerusalem M., Weinman J., Wright S., Johnston M. (1995). Generalized Self-Efficacy Scale. Measures in Health Psychology: A User’s Portfolio. Causal and Control Beliefs.

[76] Sibilia L., Schwarzer R., Jerusalem M. Italian Adaptation of the General Self-Efficacy Scale. http://userpage.fu-berlin.de/~health/italian.htm.

[77] Rosenberg M. (1965). Rosenberg Self-Esteem Scale (RSE). Accept. Commit. Ther. Meas. Packag..

[78] Prezza M., Trombaccia F.R., Armento L. (1997). La Scala Dell’autostima di Rosenberg: Traduzione e Validazione Italiana. Giunti Organ. Spec..

[79] Gori A., Craparo G., Giannini M., Loscalzo Y., Caretti V., La Barbera D., Schuldberg D. (2015). Development of a new measure for assessing insight: Psychometric properties of the insight orientation scale (IOS). Schizophr. Res..

[80] Gori A., Topino E., Svicher A., Schuldberg D., Di Fabio A. (2022). Insight orientation scale: A promising tool for organizational outcomes–A psychometric analysis using item response theory. Front. Psychol..

[81] Gosling S.D., Rentfrow P.J., Swann W.B. (2003). A very brief measure of the Big-Five personality domains. J. Res. Pers..

[82] Costa P.T., McCrae R.R. (1992). Four ways five factors are basic. Pers. Individ. Differ..

[83] Di Fabio A., Gori A., Giannini M. (2016). Analysing the psychometric properties of a Big Five measure with an alternative method: The example of the Ten Item Personality Inventory (TIPI). G. Ital. Ric. Appl..

[84] IBM Corp (2012). IBM SPSS Statistics for Windows.

[85] Arbuckle J.L. (2016). IBM SPSS Amos 24 User’s Guide.

[86] Kim H.Y. (2013). Statistical notes for clinical researchers: Assessing normal distribution (2) using skewness and kurtosis. Restor. Dent. Endod..

[87] Mulaik S.A. (2009). Foundations of Factor Analysis.

[88] Kline R.B. (2015). Principles and Practice of Structural Equation Modeling.

[89] Hooper D., Coughlan J., Mullen M.R. (2008). Structural equation modeling: Guidelines for determining model fit. Electron. J. Bus. Res. Methods.

[90] Marsh H.W., Hau K.T., Wen Z. (2004). In search of golden rules: Comment on hypothesis-testing approaches to setting cutoff values for fit indexes and dangers in overgeneralizing Hu and Bentler’s (1999) findings. Struct. Equ. Model..

[91] Marsh H.W., Hocevar D. (1985). Application of confirmatory factor analysis to the study of self-concept: First- and higher order factor models and their invariance across groups. Psychol. Bull..

[92] Byrne B.M. (2010). Structural Equation Modeling with AMOS: Basic Concepts, Applications, and Programming.

[93] Cronbach L.J. (1951). Coefficient Alpha and the internal structure of tests. Psychometrika.

[94] McDonald R.P. (2013). Test Theory: A Unified Treatment.

[95] Goldberg S.B., Hoyt W.T., Nissen-Lie H.A., Nielsen S.L., Wampold B.E. (2018). Unpacking the therapist effect: Impact of treatment length differs for high-and low-performing therapists. Psychother. Res..

[96] Firth N., Saxon D., Stiles W.B., Barkham M. (2019). Therapist and clinic effects in psychotherapy: A three-level model of outcome variability. J. Consult. Clin. Psychol..

[97] Reese R.J. (2017). The promise and challenge (and reality) of defining therapist expertise. Couns. Psychol..

[98] O’Shaughnessy T., Du Y., Davis C. (2017). Reflections on the Power to Define Psychotherapy Expertise. Couns. Psychol..

[99] Goodyear R.K., Wampold B.E., Tracey T.J., Lichtenberg J.W. (2017). Psychotherapy expertise should mean superior outcomes and demonstrable improvement over time. Couns. Psychol..

[100] Beutler L.E., Machado P.P.P., Neufeldt S.A., Bergin A.E., Garfield S.L. (1994). Therapist Variables. Handbook of Psychotherapy and Behavior Change.

[101] Beutler L.E., Malik M., Alimohamed S., Harwood T.M., Talebi H., Noble S., Wong E., Lambert M.J. (2004). Therapist Variables. Bergin and Garfield’s Handbook of Psychotherapy and Behavior Change.

[102] Betan E.J., Binder J.L. (2010). Clinical Expertise in Psychotherapy: How Expert Therapists Use Theory in Generating Case Conceptualizations and Interventions. J. Contemp. Psychother..

[103] Castonguay L., Hill C.E. (2023). Becoming Better Psychotherapists: Advancing Training and Supervision.

[104] Lingiardi V., Muzi L., Tanzilli A., Carone N. (2018). Do therapists’ subjective variables impact on psychodynamic psychotherapy outcomes? A systematic literature review. Clin. Psychol. Psychother..

[105] Black S., Hardy G., Turpin G., Parry G. (2005). Self-Reported Attachment Styles and Therapeutic Orientation of Therapists and Their Relationship with Reported General Alliance Quality and Problems in Therapy. Psychol. Psychother. Theory Res. Pract..

[106] Evers O., Schröder-Pfeifer P., Möller H., Taubner S. (2019). How do personal and professional characteristics influence the development of psychotherapists in training: Results from a longitudinal study. Res. Psychother. Psychopathol. Process Outcome.

[107] Wampold B.E., Brown G.S. (2005). Estimating variability in outcomes attributable to therapists: A naturalistic study of outcomes in managed care. J. Consult. Clin. Psychol..

[108] Budman S.H. (1981). Significant treatment factors in short-term group psychotherapy. Group.

[109] Sifneos P.E. (1981). Short-term dynamic psychotherapy: Its history, its impact and its future. Psychother. Psychosom..

[110] Orlinsky D.E., Howard I.I., Garfield S.L., Bergin A.E. (1986). Process and outcome in psychotherapy. Handbook of Psychotherapy and Behavior Change.

[111] Stein D.M., Lambert M.J. (1984). On the relationship between therapist experience and psychotherapy outcome. Clin. Psychol. Rev..

[112] Romero-Moreno A., Paramio A., Cruces-Montes S., Zayas A., Guil R. (2021). Attributed Contribution of Therapist’s Emotional Variables to Psychotherapeutic Effectiveness: A Preliminary Study. Front. Psychol..

[113] Romero-Moreno A., Paramio A., Cruces-Montes S.J., Zayas A., Gómez-Carmona D., Merchán-Clavellino A. (2021). Development and Validation of the Psychotherapeutic Effectiveness Attribution Questionnaire (PEAQ-12) in a Spanish Population. Int. J. Environ. Res. Public Health.

[114] Castonguay L.G., Boswell J.F., Constantino M.J., Goldfried M.R., Hill C.E. (2010). Training implications of harmful effects of psychological treatments. Am. Psychol..

[115] Goldberg S.B., Babins-Wagner R., Rousmaniere T., Berzins S., Hoyt W.T., Whipple J.L., Miller S.D., Wampold B.E. (2016). Creating a climate for therapist improvement: A case study of an agency focused on outcomes and deliberate practice. Psychotherapy.

[116] Gori A., Topino E., Brugnera A., Compare A. (2022). Assessment of professional self-efficacy in psychological interventions and psychotherapy sessions: Development of the Therapist Self-Efficacy Scale (T-SES) and its application for eTherapy. J. Clin. Psychol..

[117] Boswell J.F., Castonguay L.G., Wasserman R.H. (2010). Effects of psychotherapy training and intervention use on session outcome. J. Consult. Clin. Psychol..

[118] Campbell J. (2013). Becoming an Effective Supervisor: A Workbook for Counselors and Psychotherapists.

[119] Statista Number of Registered Psychologists in Australia in 2021, by Gender. https://www.statista.com/statistics/806623/australia-registered-psychologists-by-gender.

[120] Statista Number of Psychologists Employed in Norway in 2021, by Age Group and Gender. https://www.statista.com/statistics/965791/number-of-employed-psychologists-in-norway-by-age-and-gender.

[121] Statista Percentage of Women among Active Psychologists in the United States from 2007 to 2019. https://www.statista.com/statistics/963476/percentage-of-women-among-psychologists-us.

[122] Klein M.H., Mathieu-Coughlan P., Kiesler D.J., Greenberg L.S., Pinsof W. (1986). The Experiencing Scales. The Psychotherapeutic Process: A Research Handbook.

[123] Klein M.H., Mathieu P., Gendlin E.T., Kiesler D.J. (1969). The Experiencing Scale: A Research and Training Manual.

[124] Pascual-Leone A., Yeryomenko N. (2017). The client “experiencing” scale as a predictor of treatment outcomes: A meta-analysis on psychotherapy process. Psychother. Res..

[125] Johns R.G., Barkham M., Kellett S., Saxon D. (2019). A systematic review of therapist effects: A critical narrative update and refinement to Baldwin and Imel’s (2013) review. Clin. Psychol. Rev..

